# Palbociclib suppresses the cancer stem cell properties and cell proliferation through increased levels of miR-506 or miR-150 in Panc-1 and MiaPaCa-2 cells

**DOI:** 10.55730/1300-0152.2622

**Published:** 2022-07-18

**Authors:** Özge RENCÜZOĞULLARI, Elif Damla ARISAN

**Affiliations:** 1 İstanbul Kültür University, Science and Literature Faculty, Department of Molecular Biology and Genetics, İstanbul, Turkey; 2 Institute of Biotechnology, Gebze Technical University, Gebze, Turkey

**Keywords:** Pancreatic cancer, CDK4/6 inhibitor, miR-506, miR-150, miR-208, Wnt/ β-catenin, leptin

## Abstract

The prognostic characteristics of pancreatic cancer (PC) are determined by the contributing factors from the tumor microenvironment. Leptin is a critical oncogenic factor released by adipocytes as an adipokine into the tumor microenvironment, where it promotes tumor development by activating cancer stem cell (CSC) molecular regulators Notch, Hedgehog, and Wnt/β-catenin signaling. One of the downstream targets of these pathways is CDK4/6 and cyclin D which is controlled by P16 INK4A that is highly mutated in PC. Therefore, the purpose of this study was to determine the effect of a CDK4/6 inhibitor, palbociclib, on Leptin-induced PC cells and to target the Notch, Hedgehog, and Wnt/β-catenin signaling pathways via miR-150, miR-506, and miR-208 modulation. Leptin treatment increased the ability of Panc-1, MiaPaCa-2, and Capan-2 cells to proliferate and decreased the effect of palbociclib. Additionally, tumorspheres were generated from Leptin-treated (Leptin+) and Leptin-untreated (Leptin−) Panc-1 and MiaPaCa-2 cells and transfected with miR-506, miR-150 (tumorsuppressor miRNAs), or anti-miR-208 (oncomiR), followed by palbociclib treatment. Forced expression of miR-506 or miR-150 significantly increased the susceptibility of Leptin+ cells to palbociclib treatment by inhibiting colony and tumor spheroid formation, and CD44 expression in Panc-1 and MiaPaCa-2 cells. Additionally, the increased miR-150 expression is more effective at inhibiting N-cadherin, β-catenin, p-GSK3β, Notch, and Wnt5a/b expression in Leptin−/+ Panc-1 and MiaPaCa-2 cells. As a result, palbociclib suppressed the CSC profile induced by leptin treatment, inhibiting both tumorsphere forms and leptin-targeted signaling pathways, thereby disabling the Panc-1 and MiaPaCa-2 cells’ resistance mechanism. Increased expression of miR-506 or miR-150 and inhibition of miR-208 enhanced sensitivity of Panc-1 and MiaPaCa-2 Leptin−/+ cells to palbociclib treatment. As a result, this study proved that combining inhibitors of CSC molecular regulators with palbociclib improves the success rate of inhibition of PC cell proliferation.

## 1. Introduction

Pancreatic cancer, which has almost the same incidence and mortality rate and a 5-year survival rate of only 6.5%, is an aggressive type of cancer that cannot be diagnosed early (Kim et al., 2014). Although most PC risk factors such as obesity do not directly cause PC, they trigger cancer development. The mortality rate due to PC is more than 50% in obese individuals with a body mass index (BMI) higher than 30. Various hormones released from adipose tissue due to obesity and inflammatory cytokines such as adipokines trigger obesity-related cancer (Sakorafas et al., 2000). Leptin, an adipokine, is an essential factor that increases tumor cell proliferation and angiogenesis in cancer cases (Pothuraju et al., 2018). Accordingly, increased leptin triggers cancer formation by increasing insulin growth factor (IGF-1) and increasing cell growth through activation of PI3K/AKT/mTOR signaling and suppressing apoptosis (Harbuzariu et al., 2018; Pothuraju et al., 2018). A study examining the expression level of leptin, which causes increased breast cancer development showed that it accelerates the PC process by activating the Notch signaling pathway associated with tumor metastasis and CSC differentiation in BxPC-3, MiaPaCa-2, Panc-1 and AsPC-1 PC cells (Ramakrishnan et al., 2011).

Accordingly, adipokine levels and their induced signaling pathways such as Notch, Hedgehog or Wnt/β-catenin are essential therapeutic targets in the diagnosis and treatment of PC. Abnormal activation of these signaling pathways is observed in many cancer cases because the induction of drug resistance mechanisms and metastasis process, especially by triggering CSC differentiation (Nusse and Clevers, 2017), (Matsui, 2016). Therefore, targeting these signaling pathways in suppressing tumor growth is a therapeutic target that will prevent drug resistance mechanisms, metastasis, and cell proliferation signals; moreover, it will yield valuable results for clinical trials. Pancreatic CSCs are one of the most important factors hindering the PC therapy process. CD133/CXCR4, PD2/Paf1, CD44/CD24/ ESA, c-MET, and ALDH1 are pancreatic CSC markers and their expression levels are quite high in drug-resistant cell populations. The drug combinations of cyclopamine, rapamycin, and gemcitabine reduced cell survival through targeting CSCs (Chen et al., 2013).

An important reason why the incidence of PC and the death rate due to PC are very close to each other is because PCs are generally metastatic tumors. According to studies, pancreatic CSCs and epithelial-mesenchymal transition (EMT) are interrelated with leptin that is an important factor in the tumor microenvironment that accelerates tumor development by triggering CSC molecular regulators, Notch, Hedgehog, Wnt/β-catenin (Harbuzariu et al., 2018). The target of these signaling pathways, cyclin D1, is expressed at high levels in PC cells (Vaz et al., 2014). Therefore, it is predicted that an inhibitor targeting cyclin D1 may be helpful in the treatment of aggressive PC. Palbociclib, a CDK4/6 inhibitor, is approved by the FDA and used in advanced postmenopausal breast cancer therapy. In PC cases, cell proliferation due to high cyclin E1 can be suppressed by the CDK4/6 inhibitor palbociclib (Franco et al., 2014). Recent studies have shown that microRNAs (miRNAs) play a role in cell proliferation, stem cell differentiation, adipogenesis and metastasis by regulating stem cell markers such as c-Met and ALDH1 in normal and cancer cells (Guo et al., 2018; Guo, Xu, et al., 2018) ). According to the information obtained from miRNA platforms, miR-150 as a tumor suppressor miRNA, targets β-catenin, which plays vital role in EMT, and PPARGC1α genes that function in adipose tissue (Li et al., 2019). Another study showed that miR-506 triggered autophagy-related death by targeting the STAT3 gene of PC cells (Zhou et al., 2019). According to the data we obtained from miRNA platforms, Notch and vimentin are among the target genes of miR-506 (Arora et al., 2013). Another study stated that miR-208, an oncomiR, acts as an obesity trigger in mouse cardiomyocyte cells and its expression decreases in the presence of mTOR inhibitor rapamycin, while weight loss increases despite leptin resistance (Gul et al., 2015).

In this study, CSC phenotype of Panc-1 and MiaPaCa-2 PC cells were induced by leptin treatment which increased the colony forming potential by 50% and these cells expressed the Notch and the mesenchymal markers such as CD44, N-cadherin, Vimentin higher than control cells. A unique value of our study is to investigate the effect of palbociclib on these cells and targeting Notch, Hedgehog and Wnt/β-catenin signaling pathways through modulation of miR-150, miR-506 and miR-208. According to the results obtained, palbociclib suppressed the CSC features and cell proliferation due to increased level of miR-506 or miR-150 in leptin−/+ Panc-1 and MiaPaCa-2 cells.

## 2. Materials and methods

### 2.1. Cell culture

MiaPaCa-2 (ATCC^®^ CRL-1420TM, RRID: CVCL_0428), Panc-1 (ATCC^®^ CRL1469TM, RRID: CVCL_0480) and Capan-2 (ATCC^®^ HTB-80™, RRID: CVCL_0026) pancreatic cancer cell lines were incubated in Dulbecco’s modified Eagles medium (GIBCO-Life Technologies, Carlsbad, CA, USA) with 10% fetal bovine serum (FBS) (Pan Biotech GmbH, Aidenbach, Germany) and 1% penicillin/ streptomycin (Pan Biotech GmbH, Aidenbach, Germany) in 37 °C with 5% CO_2_. Leptin treatment was carried out in such a way that 1.2 nM (40 ng/mL) recombinant leptin (level in PC patient serums) (Peprotech Cat. No: 300-27, Hamburg, Germany) was incubated for 24 h after 16-h serum starvation (without FBS) of PC cells (Ekmen et al., 2016) and cells were continuously treated with the certain dose of leptin (Fan et al., 2015). These cells were named as leptin+ cells. Transfection of miR-506 or miR-150 mimic and anti-miR-208 was proceeded as described previously (Rencuzogulları et al., 2021). Briefly, Panc-1, MiaPaCa-2, and Capan-2 PC cells with/without leptin were transfected with a 1:3 lipogenic transfection agent, miRNA mimic 5 nM (miRCURY LNA miRNA Mimics, Cat. No: 339173; Qiagen, Hilden, Germany) and anti-miR 50 nM (miRCURY LNA miRNA Inhibitors, Cat. No: 339121; Qiagen, Hilden, Germany). mirVana™ miRNA Mimic, Negative Control (Cat. No: 4464058; Thermo Scientific, Massachusetts, USA), and Anti-miR™ miRNA Inhibitor Negative Control (Cat. No: AM17010 Thermo Scientific, Massachusetts, USA), which had no identifiable effects on known miRNA function, were used to control transfection quality and evaluation of experimental miRNA mimic/ anti-miR on target gene expression. After the transfection agent was kept in medium without FBS for 5 min (min) at room temperature, it was combined with the medium containing mimic or anti-miR and incubated for 25 min at room temperature. Cells were incubated with transfection solution for 48 h.

### 2.2. MTT cell viability assay

1× 10^4^ leptin- and leptin+ PC cells were seeded into 96-well plates. Following overnight incubation time, the cells were exposed to 2 and 3 μM palbociclib (10 mM main stock, Selleckhem, Houston, USA) for 24 h. Then 10 μL of 2,5-diphenyl-2H-tetrazolium bromide (MTT) reagent (5 mg/mL) was added to each well and incubated for 4 h at 37 °C with 5% CO_2_. Formazan crystals were dissolved with 100 μL of DMSO and analyzed with an Elisa reader at 570 nm. MTT assay was performed at least three times with four repeats.

### 2.3. Colony formation assay

Leptin treatment was performed as previously mentioned as 1.2 nM leptin for 24 h following 16 h serum-starvation. At that point, miRNA mimic and anti-miRNA transfections were proceeded for leptin+ and leptin-cells. The cells were seeded into 6-well plate at 3 x 10^3^ cells/well and then they were exposed to 3 μM palbociclib treatment. Colony assay was then performed as described previously (Rencuzogulları et al., 2020). Briefly, drug containing media was removed over the cells and fresh media was added. Then, the cells were incubated until untreated control group reached 80% confluence of well following treatments. Following fixation, the cells were stained with 0.5% crystal violet. Colonies were visualized and counted by ImageJ analysis program.

### 2.4. miRNA analysis

5×10^5^ cells were seeded into 6-well plates. Following overnight incubation time, the cells were transfected with miR-506, miR-150, and anti-miR-208 for 48 h, as mentioned in part 2.1. Then the cells were treated with 3 μM Palbociclib for 24 h. According to the manufacturer’s instructions, total miRNA was extracted by using miRNeasy (Qiagen). The cDNA amplification was proceeded by miScript RT kit (Qiagen). The accuracy of transfection of mimic miR-506 or miR-150 and anti-miR-208 was controlled by measuring the expression of mature miRNAs with quantitative real-time PCR (qRT-PCR) with miScript SYBR Green PCR Kit (Qiagen). The human noncoding small nuclear RNA U6 was used as the endogenous control to normalize the obtained miRNAs. The primers for qRT-PCR: miR-506 5′-CCTTGGCACCCTTCTGTAGA-3′; miR-150: 5′-GAGAGACGCATAAAAGCCGC-3′; miR-208: 5′-ATAAGACGAGCAAAAAGCTTGT-3′ U6-F: 5′-TGGCACCCAGCACAATGAA-3′; U6-R: 5′-CTAAGTCATAGTCCGCCTAGAAGCA-3′. The amplification reactions were performed in triplicate using the following cycle: 10 min at 95 °C, followed by 40 cycles of 15 s at 95 °C and 1 min at 60 °C. The Cq values were calculated using the Bio-Rad CFX Manager System software. The relative fold change for each miRNA was calculated using the comparative Cq (2^−ΔΔCq^) method.

### 2.5. Annexin V/PI staining and cell cycle analysis

Apoptotic cells were examined after cells were stained by Annexin V and propidium iodide (PI), by flow cytometer analysis using C6 software (BD Bioscience). Following treatments of Panc-1 and MiaPaCa-2 cells were harvested by trypsin-EDTA. 10^4^ cells were gated and analyzed with FL1(488 nm) and FL3 (640 nm) filters. Cell cycle analysis was performed by PI staining after cells were fixed with 70% Ethanol. Flow cytometric analysis was continued for 10^4^ cells with FL3 filter (BD Accuri).

### 2.6. Immunofluorescence analysis

Immunofluorescence analysis was performed to confirm the CD44 level by florescence microscopy. Panc-1 and MiaPaCa-2 cells transfected with miR-506 mimic, miR-150 mimic, and anti-miR-208 were seeded at 5×10^4^ cells on a coverslip and allowed to adhere overnight. After 24 h of applying 3 μM palbociclib, the medium on the cell was gently removed. After washing with 1X PBS, the cells were fixed at room temperature for 20 min with methanol/acetone (1:1) fixative at −20°C. 3% BSA was used as the blocking solution to prevent nonspecific binding. Next, the cells were incubated with CD44 primary antibody (prepared 1:1000 with 3% BSA) at room temperature for 2 h and then they were washed 3 times with 1X PBS containing 0.01% Triton-x to eliminate excess binding. It was incubated with an Alexa-488-conjugated antimouse antibody as secondary antibody for 2 h at room temperature. The cells were observed under a fluorescence microscope. The total fluorescence integrity was quantitatively calculated and analyzed by the Image J method calculated by the formula that corrected total cell fluorescence = integrated density – (area of selected cell × mean fluorescence of background readings.

### 2.7. Wound healing assay

The effect of palbociclib on cell migration in MiaPaCa-2 and Capan-2 PC cells was investigated with wound healing test, which is a simple and inexpensive method. When PC cells and cells transfected with miR-506 mimic, miR-150 mimic, and anti-miR-208 reached 80% density, 3 μM palbociclib was applied and then the cells were incubated in medium containing 1% FBS overnight. The 6-well plate with the cells was drawn with the 10 μm tip. The cells were washed with 1X PBS and then incubated with treated and nontreated media. However, the media used in this assay contained %5 FBS in order to facilitate cell migration. Wound area was visualized at 0 and 48 h with a light microscope and with DiOC6 staining and the migration values of cells were calculated by taking the average values of the images recorded from 5 different regions.

### 2.8. Soft agar assay

The effect of Panc-1 and MiaPaCa-2 cells that acquired CSC profile by leptin application on the 3D tumorsphere structure of palbociclib after miRNA mimic, and anti-miR transfection was analyzed. Firstly, miR-506 mimic (5 nM), miR-150 mimic (5 nM) and anti-miR-208 (50 nM) were transfected to leptin−/+ Panc-1 and MiaPaCa-2 cells for 48 h and grown in soft agar for 14 days at 37 °C. To form the ground layer, soft agar consists of two layers. 0.5% agarose, 20% FBS, and 2% Pen./Strep. It was mixed with 2X DMEM containing 1:1 ratio. The 6-well plate was homogeneously coated with 1 mL of basement solution. The upper gel solution containing 0.3% agarose and 2X DMEM containing 2.5×10^4^ cells/mL is prepared at a ratio of 1:1, and 3 μM palbociclib was added to the top layer solution at this stage. In order to prevent the agarose gel from drying, 500 μL of medium with or without drug was added to the top. Colony diameters were examined at the end of the 14th day. Colony diameters were compared between leptin-treated and non-leptin-treated cells, and statistical analysis was performed.

### 2.9. CD44, CD144, and CD24 analysis

Leptin- and leptin+ PC cells were seeded at 6×10^5^ cells/well then transfected with miR-506 mimic, miR-150 mimic, and anti-miR-208 for 48 h and then 3 μM palbociclib was applied for 24 h. The cells were blocked with 1% BSA for 30 min at room temperature. They were then washed with 1X PBS containing 0.01% triton-x and then incubated at room temperature for 1 h with fluorescent monoclonal antibodies for CSC markers (CD24+, CD44+, CD133+ conjugated with PE, APC, and FITC, respectively) prepared at 1:1000 in BSA. Cells incubated with primary antibodies and unstained control groups were analyzed for 10^4^ cells by cell flow cytometry (BD Bioscience). The cells stained with CD44 only were determined on the dot plot with the SSC and FL-1 (488 nm) filter. The drug-free control and other cell groups could be compared and analyzed according to this limit. Boundaries were set with SSC with FL-2 (565 nm) filter for CD133 and with SSC with FL-3 (640 nm) filter for CD24.

### 2.10. Immunoblotting

Total protein isolation was performed from leptin- and leptin+ Panc-1 and MiaPaCa-2 ceflls after miR-506 mimic and miR-150 mimic transfections and 3 μM palbociclib treatment as described previously (Berrak et al., 2016). Primary antibodies were used as 1:1000 dilution and secondary antibodies were used with 1:3000 dilution. N-cadherin, β-Catenin, p-GSK-3β (S9), Dvl-2 Wnt5a/b, Notch-1, and CD44 protein expressions were investigated in Panc-1 and leptin+ Panc-1 cells. Protein expressions of p-AKT (S473), E-cadherin, β-Catenin, Vimentin, Dvl-2, Notch-1, and Snail in MiaPaCa-2 and leptin+ MiaPaCa-2 cells were investigated. It was analyzed by normalizing according to β-actin levels with the Image J program.

### 2.11. Statistical analysis

GraphPad software (4.04 version) was used for the analysis of at least 2 replications of the experiment (RRID:SCR_000306). MTT cell viability, colony formation analysis, wound healing assay, soft agar assay, and flow cytometric analysis was proceeded by three biological repeats. The results were statistically analyzed by Bonferroni’s multiple comparison analysis method. Immunoblotting results were calculated numerically with the Image J (RRID:SCR_003070) analysis program and statistical data were obtained with 2-way ANOVA Tukey’s multiple comparison analysis by normalizing each band profile according to its β-actin values.

### 2.12. List of antibodies

Primary antibodies N-cadherin, E-cadherin, Vimentin, β-catenin, p-GSK3β (S9), Dvl-2, Wnt5a/b, Notch1, CD44, p-Akt (ser473), Snail, and actin were purchased from Cell Signaling Technology (CST; Danvers, MA). CD44, CD133, and CD24 antibodies were purchased from Biolegend (BioLegend, Inc., San Diego, USA). Secondary Alexa 488-conjugated antibody and HRP conjugated antibody were purchased from CST.

## 3. Results

### 3.1. Palbociclib suppressed the colony forming potential of Panc-1 and MiaPaCa-2 cells, which increased with leptin treatment

**Figure 1:**
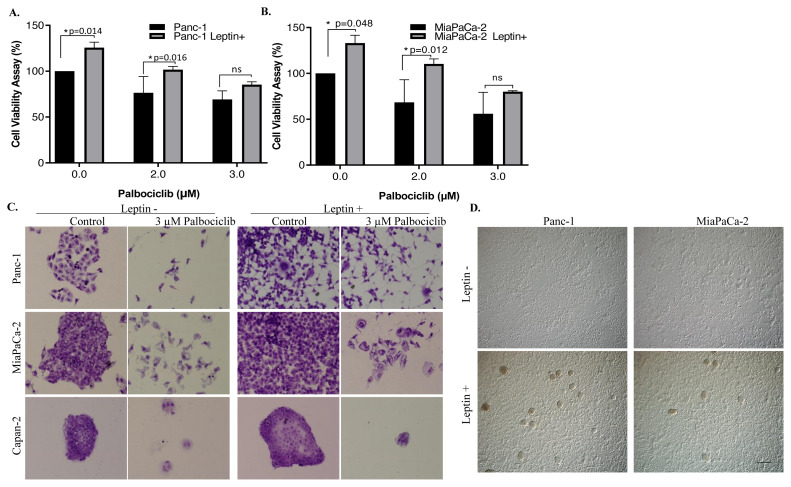
Leptin reduced the sensitivity of pancreatic cancer cells to palbociclib (PD) treatment. **A–B.** 40 ng/mL leptin was applied to Panc-1 (A) and MiaPaCa-2 (B) cells. MTT cell viability test was performed after 24 h of 3 μM palbociclib treatment. Four replicates of 2 different experiments mean/SD analysis was proceeded. **C.** The long-term effect of PD on leptin treated (leptin+) and un-treated (leptin −) Panc-1, MiaPaCa-2 and Capan-2 cells was analyzed by colony formation assay. **D.** Representation of cell clusters in leptin-treated cells. When leptin-treated Panc-1 and MiaPaCa-2 cells reached approximately 90% density. 10× magnification. Scale bar is 100 μm.

It was determined that the cell proliferation rate of Panc-1 and MiaPaCa-2 cells treated with leptin increased and accordingly, it caused an increase in the relative cell viability by 25% and 33%, respectively. The viability of Panc-1 and Panc-1 Leptin+ cells were 76% and 101% after treatment with 2 μM palbociclib, respectively, whereas it was 69% and 85% following 3 μM palbociclib treatment, respectively (Figure 1A). The viability of MiaPaCa-2 and MiaPaCa-2 Leptin+ cells treated with 2 μM palbociclib was 68% and 110%, whereas it was 55% and 80% following 3 μM palbociclib treatment, respectively (Figure 1B). As a result, it was determined that 3 μM palbociclib caused a significant decrease in the viability of the cells with or without leptin treatment. As a result of the colony formation experiment, 20× magnified images obtained by a light microscope of cells stained with crystal violet were shown (Figure 1C). By leptin application, the diameters of Leptin+ Panc-1, MiaPaCa-2, and Capan-2 cell colonies expanded. Another significant issue is that Panc-1 and MiaPaCa-2 cell morphologies were changed by leptin application. Accordingly, the cells shrunk and had prominent fibroblast-like extensions. Colony-forming potential of leptin+ cells was significantly suppressed by the treatment of 3 μM palbociclib. Leptin treatment was repeated every 4 days and light microscopy images were taken at ten-fold magnification of Panc-1 and MiaPaCa-2 cells, which reached 90% confluence. According to the results obtained, cell clusters like stem cell spheroid structures (Vazquez et al., 2017) were observed in the leptin-administered groups (Figure 1D).

### 3.2. Palbociclib modulated the intracellular level of miR- 506, miR-150, and miR-208 in leptin−/+ Panc-1 and MiaPaCa-2 cells

**Figure 2:**
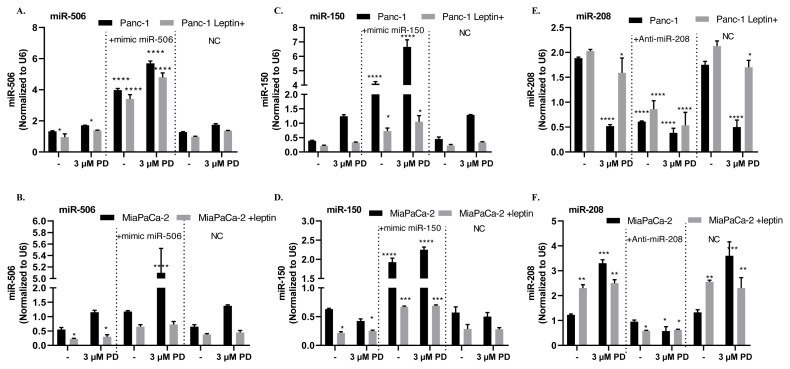
Palbociclib treatment increased the tumor suppressor miR-506 and miR-150 levels in both Panc-1 and MiaPaCa-2 cells**. A–F.** The effect of PD on the indicated miRNA expressions in leptin+/− Panc-1 and MiaPaCa-2 cells after miR-506 and miR-150 mimic and anti-miR-208 transfection. The levels of miR-506 (A–B), miR-150 (C–D), and miR-208 (E–F) were determined by using real-time PCR in leptin+/− Panc-1 and MiaPaCa-2 cells. Normalization was performed with the internal endogenous control RNU6. The results obtained are the mean/SD of the 2-replication data of two different experiments. NC: Negative control A. * p < 0.05 (nontreated control vs. leptin+; non-treated control vs*.* 3 μM PD-treated leptin− and leptin+ cells*)*, ****p < 0.0001 (nontreated control vs. mimic miR-506-transfected leptin− and leptin + cells), B. *p < 0.05 (nontreated control vs*.* leptin+; nontreated control vs*.* 3 μM PD-treated leptin+ cells), C. *p < 0.05 (nontreated control vs. mimic miR-150-transfected leptin + cells) ****p < 0.0001 (nontreated control vs*.* mimic miR-150-transfected leptin− cells), D. * p < 0.05 (nontreated control vs*.* leptin+; nontreated control vs. 3 μM PD-treated leptin− and leptin+ cells*)*, ****p < 0.0001 (nontreated control *vs.* mimic miR-506-transfected leptin− cells), E. *p < 0.05 (nontreated control *vs.* 3 μM PD-treated leptin+ cells) **** p < 0.0001 (nontreated control vs*.* 3 μM PD-treated leptin− cells; nontreated control *vs.* anti-miR-208 transfected leptin− and leptin + cells), F. **p < 0.01 (nontreated control vs*.* leptin+ cells; nontreated control vs*.* 3 μM PD-treated leptin + cells) *p < 0.05 (nontreated control vs*.* anti-miR-208 transfected leptin + cells; non-treated control vs*.* 3 μM PD-treated leptin − and leptin + cells).

To understand the regulatory role of palbociclib on miRNA expression, under leptin-mediated responses in Panc-1 and MiaPaCa-2 cells, miR-506 and miR-150 mimic miRNAs and anti-miR-208 were transfected. Moreover, negative controls (NC) which had no identifiable effects on known miRNA function were used to control transfection quality and evaluation of experimental miRNA mimic/anti-miR on target gene expression. In this study, the expression levels of the indicated miRNAs in leptin-treated cells were also normalized according to the intracellular endogenous control miRNA, RNU6 (Figure 2). The miR-506 level decreased 1.6-fold (p < 0.05) in leptin-treated Panc-1 cells compared to the control group and increased when 3 μM palbociclib was treated. After miR-506 mimic transfection, miR-506 level increased 4- and 3.6-fold in Panc-1 and Panc-1 leptin+ cells, respectively, whereas miR-506 expression increased 5.7- and 4.8-fold, respectively, with palbociclib treatments (****p < 0.0001) (Figure 2A). We determined that miR-506 level was lower in MiaPaCa-2 cells than in Panc-1 cells. When leptin was treated, this level decreased approximately 2 times (p < 0.05). Palbociclib treatment did not significantly differ in miR-506 level in leptin+ MiaPaCa-2 cells. However, palbociclib treatment increased miR-506 expression 10-fold (****p < 0.0001) in miR-506 mimic transfected cells, while there was no difference observed in leptin+ cells (Figure 2B). miR-150 expression, which was expressed in low amounts in Panc-1 cells, was further decreased by leptin treatment. miR-150 mimic transfection increased 10 times (****p < 0.0001) in Panc-1 cells and 1.5 times (*p < 0.05) in Panc-1 leptin+ cells compared to the control group. With Palbociclib treatment, 16-fold and 4-fold increase was observed in miR-150 mimic transfected Panc-1 and Panc-1 leptin+ cells compared to the control group, respectively (p < 0.0001) (Figure 2C). miR-150 expression was found to be 3 times lower in leptin-treated cells compared to the control group. MiR-150 expression increased 3.2-fold and 3-fold, respectively, in MiaPaCa-2 and MiaPaCa-2 leptin+ cells with miR-150 transfection. Palbociclib treatment increased miR-150 expression by 3.6-fold compared to the control group in miR-150 mimic-transfected MiaPaCa-2 cells; however, no significant difference was observed in leptin+ cells (Figure 2D). Leptin caused an increase in miR-208 level. With Palbociclib application, miR-208 level decreased 4-fold, but this decrease was only 1.3-fold in leptin+ cells. With anti-miR-208 transfection, miR-208 expression was significantly reduced in Panc-1 leptin−/+ cells. A higher suppression of miR-208 expression was observed when palbociclib was applied to the same cells (****p < 0.0001). However, it was noted that miR-208 expression was 1.5 times higher when leptin+ Panc-1 cells were compared with leptin-Panc-1 cells (Figure 2E). miR-208 expression increased 2.1-fold with leptin treatment to MiaPaCa-2 cells, as in Panc-1 cells **p < 0.02). However, palbociclib caused a 3-fold (***p < 0.001) and 2-fold (**p < 0.02) increase in miR-208 expression in MiaPaCa-2 and leptin+ cells compared to the control group, respectively. Anti-miR-208 caused a significant decrease of miR-208 levels in leptin+/− MiaPaCa-2 cells (Figure 2F).

### 3.3. Modulation of miR-506, miR-150, and miR-208 levels affected the apoptotic response of Panc-1 and MiaPaCa-2 cells to Palbociclib treatment

**Figure 3:**
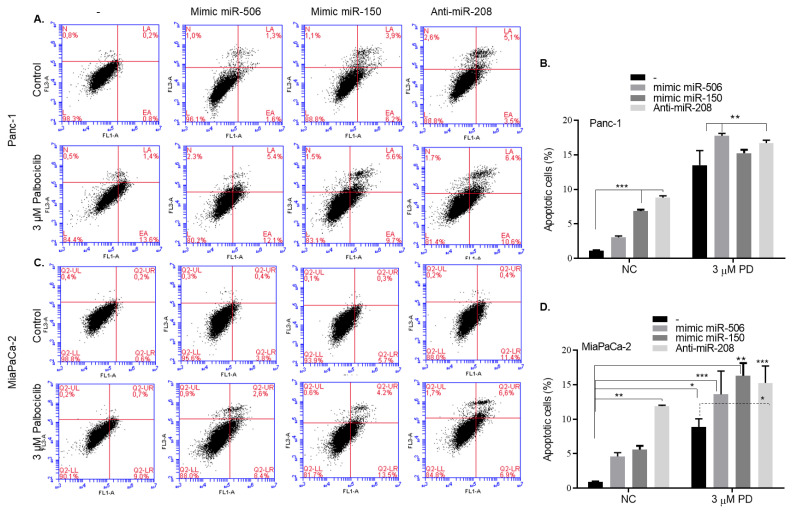
miR-506, miR-150 mimic, and anti-miR-208 transfections increased apoptotic effect of palbociclib in Panc-1 and MiaPaCa-2 cells. **A–D.** Cells were exposed to 3 μM palbociclib for 24 h following the transfection protocol. AnnexinV/PI− stained Panc-1 (A–B) and MiaPaCa-2 (C–D) cells were analyzed by flow cytometry. The results obtained are the mean/SD of two different experiments. NC: Negative control B. **p < 0.01 (3 μM PD-treated vs*.* miR-506-transfected and antimir-208-transfected), ***p < 0.001 (nontreated control vs. mimic miR-506 or anti-miR-208-transfected) D. *p < 0.05 (nontreated control vs*.* 3 μM PD-treated; 3 μM PD-treated vs. anti-miR-208-transfected), **p < 0.01 (nontreated control vs*.* anti-miR-208-transfected; nontreated control vs*.* 3 μM PD-treated-mimic miR-150-transfected), ***p < 0.001 (nontreated control vs*.* 3 μM PD-treated-mimic miR-506-transfected; nontreated control vs. 3 μM PD-treated-anti-miR-208-transfected).

The effects of miR-506, miR150 mimic and anti-miR-208 transfections in Panc-1 and MiaPaCa-2 cells on the apoptotic cell death of palbociclib were examined by cell flow cytometry with AnnexinV/PI staining (Figures 3A–3D). Apoptotic cell death was increased after palbociclib treatment to 17% in miR-506 transfected-Panc-1 cells and to %15 in miR-150 transfected-Panc-1 cells (Figure 3A–B). This ratio was analyzed respectively as 10% and 17% in MiaPaCa-2 cells (Figures 3C and 3D). Therefore, forced expression of miR-150 increased sensitivity of Panc-1 and MiaPaCa-2 cells to palbociclib treatment. Moreover, the downregulation of miR-208 enhanced the apoptotic effect of palbociclib in both cell lines. The effect of palbociclib on cell cycle after miRNA transfections was investigated by flow cytometry after propidium iodide staining in Panc-1 and MiaPaCa-2 cells (Figure S1). No significant difference was observed in the effect of palbociclib on cell cycle after miR-506 and miR-150 mimic transfection into Panc-1 and MiaPaCa-2 cells. When palbociclib was applied to cells that transfected with miR-506 mimic, an increase of 6% was observed in the SubG1 population in both Panc-1 and MiaPaCa-2 cells. Suppression of miR-208 increased cells in G1 phase by 5%. However, when palbociclib was applied to these cells, it was determined that 58% of the cells remained in the G2/M phase in Panc-1 cells.

### 3.4. Upregulation miR-150 caused significant decrease of migration potential of MiaPaCa-2 cells following palbociclib treatment

**Figure 4:**
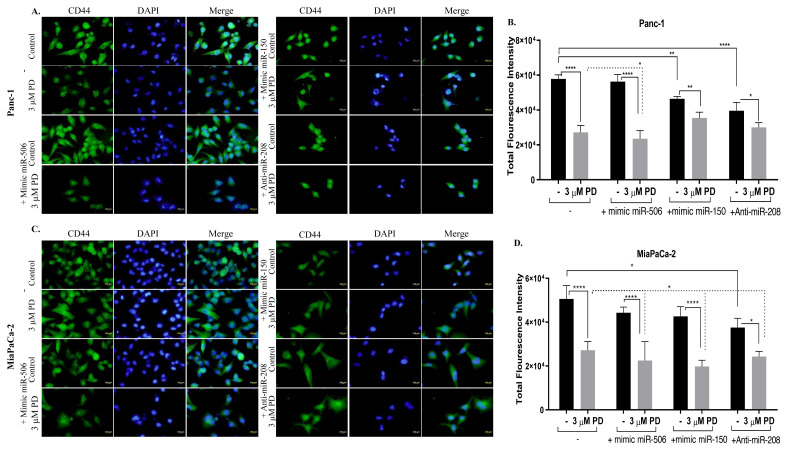

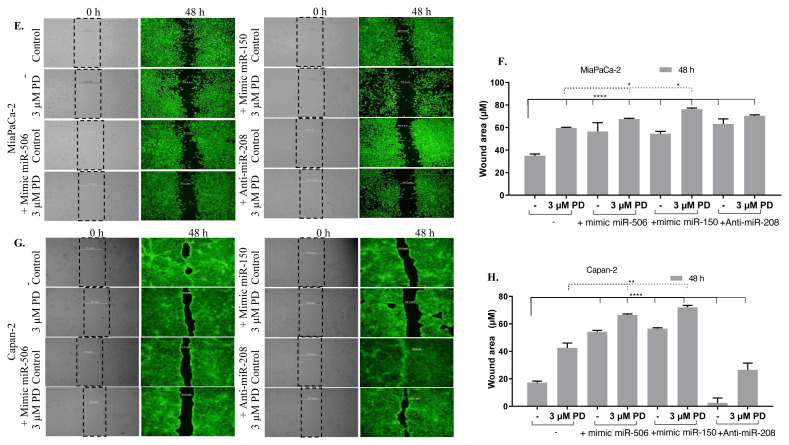
Increased expression of miR-506 or miR-150 reduced the metastatic potential of pancreatic cancer cells**. A–D.** Effect of palbociclib on CD44 expression in Panc-1 (A–B) and MiaPaCa-2 (C–D) cells with increased miR-506, miR-150 expression and inhibited miR-208 expression was determined by immunofluorescence assay. B. CD44 expression was analyzed with the Image J program by taking the total fluorescent intensity values of the green fluorescence. In order for the decrease in cell numbers not to change CD44 expression, the obtained cell brightness results were obtained by dividing by the number of nuclei (DAPI). *p < 0.05, **p < 0.01, ***p < 0.001, ****p < 0.0001. **E–H.** Effect of miR-506, miR-150, and miR-208 on the migration potential of palbociclib-treated MiaPaCa-2 (E–F) and Capan-2 (G–H) cells was analyzed by wound healing assay. Wound closure was visualized by fluorescence microscopy after DiOC6 staining. Data presented is mean ± SD of at least three separate experiments * p < 0.05 (MiaPaCa-2: 3 μM PD treated vs. 3 μM PD+ miR-506; 3 μM PD treated vs. 3 μM PD+ miR-150), **p < 0.02 (Capan-2: 3 μM PD treated vs. 3 μM PD+ miR-506; 3 μM PD treated vs. 3 μM PD+ miR-150, **** p < 0.0001 (nontreated control vs. treated groups).

CD44 is a transmembrane cell surface receptor that acts as a tumor initiating factor which PC has a high level in stem cell profiles and is an important therapeutic target. Immunofluorescence technique was used to determine the effect of palbociclib on CD44 expression in Panc-1 and MiaPaCa-2 cells modulated for miR-506, miR-150 and miR-208 (Figures 4A–4D). CD44 levels displayed as green fluorescent dots were analyzed quantitatively by calculating the total fluorescence brightness. The result was normalized by dividing by the number of cells so that the decreased cell number with palbociclib and miRNA treatments did not affect the CD44 expression. CD44 expression was significantly decreased by palbociclib treatment in Panc-1 (Figures 4A and 4B) and MiaPaCa-2 (Figures 4C and 4D) cells (****p < 0.0001). When intracellular miR-506 or miR-150 level was increased, it was determined that CD44 level decreased more with palbociclib treatment in Panc-1 and MiaPaCa-2 cells (*p < 0.05). CD44 level showed a significant decrease when intracellular miR-208 was inhibited in both Panc-1 and MiaPaCa-2.

Wound healing assay was performed to examine the effects of miR-506, miR-150 and miR-208 on the migration potential of palbociclib-treated MiaPaCa-2 (Figures 4E and 4F) and Capan-2 cells (Figures 4G and 4H). The results obtained were graphed by taking the average of the measurements made from five different regions. Compared with the control group, an increase of intracellular miR-506 and miR-150 was observed; however, suppression of miR-208 decreased the migration potential of MiaPaCa-2 cells. The wound healing suppressive effect of palbociclib was significantly increased when the intracellular miR-150 level was increased, while a decrease in cell viability was also observed (*p < 0.05). When palbociclib was applied to miR-208-inhibited MiaPaCa-2 cells, it was also determined that the migration capacity of the cells was reduced. However, when miR-208 was inhibited, wound healing of Capan-2 cells was accelerated and the wound was closed entirely.

### 3.5. The colony-forming potential of Panc-1 and MiaPaCa-2 cells increased by leptin treatment was suppressed by miR-506 mimic, miR-150 mimic and anti-miR-208 transfections

**Figure 5:**
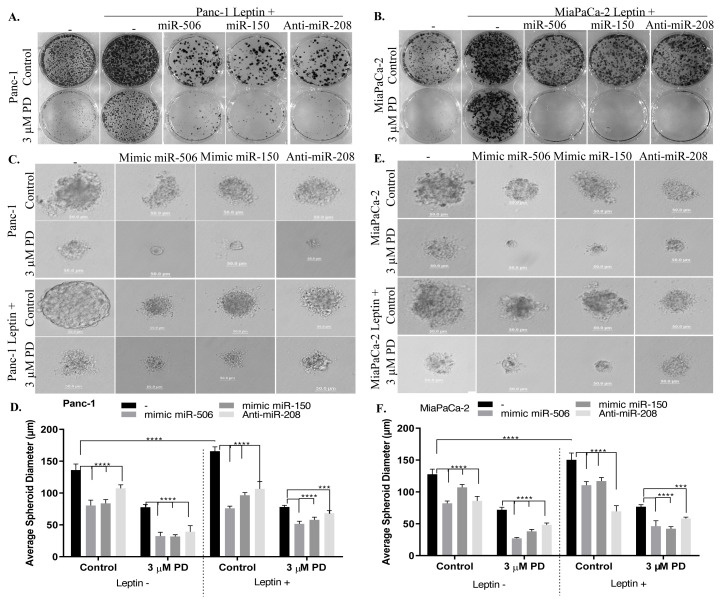

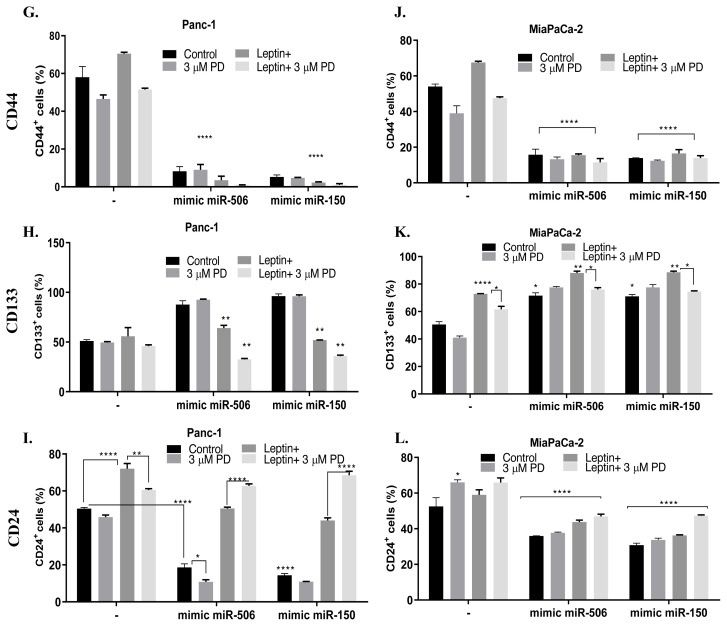
Modulation of miR-506, miR-150, and miR-208 led to significant reduction of tumor spheroid forms of palbociclib-treated cells. **A–B.** The effect of miRNA modulations on the colony forming potential of the leptin+ Panc-1 (A) and MiaPaCa-2 (B) cells following palbociclib (3 μM PD) treatment. **C–F**. The effect of miR-506, miR-150, and miR-208 expressions on anchorage-independent growth in the presence and absence of palbociclib in leptin+/− Panc-1 (C–D) and MiaPaCa-2 (E–F) cells were analyzed by soft agar assay. It was visualized using a light microscope at the end of the 14-day incubation period. A graph was created by taking the average of the diameters of 5 different tumorsphere structures. ***p < 0.001, ****p < 0.0001. **G–L.** The effect of palbociclib on the expression levels of CD44, CD133, and CD24 were analyzed with flow cytometry following modulation of mir-506, miR-150, or miR-208 in leptin+/− Panc-1 and MiaPaCa-2 cells. CD44 levels Panc-1 (G) and MiaPaCa-2 (J), CD133 levels Panc-1 (H) and MiaPaCa-2 (K) and CD24 levels Panc-1 (I) and MiaPaCa-2 (L). Data presented is mean ± SD of at least three separate experiments * p < 0.05, **p < 0.02, **** p < 0.0001.

Colony numbers and colony diameters were enlarged in leptin-treated Panc-1 (Figure 5A) and MiaPaCa-2 (Figure 5B) cells. When the expression of miR-506 or miR-150 was increased in leptin+ Panc-1 and MiaPaCa-2 cells, a decrease was observed in the number of colonies compared to both control and leptin+ cells, and a significant decrease was observed in the number of colonies when palbociclib was applied to these cells. Anti-miR-208 and palbociclib treatment synergistically reduced the colony numbers of leptin+ Panc-1 and MiaPaCa-2 cells. A striking image is the colony diameter width of the transfected Panc-1 leptin+ cells. Compared to the control group, it was observed that the colony numbers were lower, but the colony diameters were larger. It was determined that the increase of miR-506, miR-150 expression, and inhibition of miR-208 expression significantly suppressed the colony forming potential of both Panc-1 leptin+ and MiaPaCa-2 leptin+ cells. In addition, changing miRNA expressions produced a synergistic response with palbociclib, with colony numbers significantly reduced compared to control groups. The question arose whether miR-506, miR-150, and miR-208 would give the same answer when leptin+ cells form three-dimensional (3D) tumorsphere structures. For this, soft agar experiment was carried out (Figures 5C–5F). Tumorsphere structures of Panc-1 (Figures 5C and 5D) and MiaPaCa-2 (Figures 5E and 5F) cells were significantly suppressed by the increase of intracellular miR-506 and miR-150 or by inhibition of miR-208 compared to the control group. Moreover, the extensive reduction in tumor diameters with palbociclib was observed. Tumorspheric structures gained a homogeneous and spherical structure with leptin treatment; however, when miR-506 and miR-150 expressions increased, respectively, tumorigenicities of Panc-1 and MiaPaCa-2 leptin+ cells were suppressed and their sphere diameters decreased significantly. This effect was further increased with palbociclib treatment to the same cells (****p < 0.0001).

### 3.6. Palbociclib and miR-506 or miR-150 mimic treatment in Panc-1 and MiaPaCa-2 cells decreased CD44 expression, but CD133 and CD24 CSC membrane receptors caused different responses in their expression

The effects of miR-506 or miR-150 mimic and palbociclib treatment which significantly suppress tumorsphere formation of leptin−/+ Panc-1 (Figures 5D, 5G, and 5H) and MiaPaCa-2 (Figures 5J–5L) cells, on CD44 (Figures 5G–5J), CD133 (Figures 5H–5K), and CD24 (Figures 5I–5L) membrane receptors were investigated by cell flow cytometry. CD44 expression was detected over 50% in both leptin−/+ Panc-1 and MiaPaCa-2 cells. Additionally, leptin led to significant increase of CD44 levels in Panc-1 and MiaPaCa-2 cells. Although this rate decreased with palbociclib alone, the increase of miR-506 and miR-150 expressions in the cell significantly decreased CD44 expression for both cell lines. Increased miR-506 and miR-150 caused an upregulation in CD133 level in Panc-1 and MiaPaCa-2 cells. This rate is 30% and 35% less in miR-506 and miR-150 mimic transfected Panc-1 leptin+ cells, respectively (**p < 0.02). Although palbociclib did not affect CD133 level in Panc-1 cells, CD133 levels was decreased in miR-506 and mimic-transfected Panc-1 leptin+ cells by 20% and 15%, respectively, compared to the control group (**p < 0.02). It was determined that there was a 22% increase in CD133 level with leptin in MiaPaCa-2 cells. However, CD133 level in MiaPaCa-2 leptin−/+ cells decreased by 10% with palbociclib. In MiaPaCa-2 cells transfected with miR-506 and miR-150 mimics, CD133 level increased by 20% and 16%, respectively, compared to control groups (*p < 0.05). In addition, CD133 levels increased by 12% and 14% in miR-506 or miR-150 mimic transfected-MiaPaCa-2 leptin+ cells, respectively. However, palbociclib decreased CD133 level by approximately 7% in these cells (*p < 0.05). CD24 expression was 70% in Panc-1 leptin+ cells and this rate decreased by 10% with palbociclib (**p < 0.02). The increase in miR-506 and miR-150 expressions downregulated the CD24 levels in Panc-1 cells by 40% (****p < 0.0001). In addition, CD24 levels decreased to 16% (*p < 0.05) when these cells were exposed to palbociclib. CD24 level decreased by 22% and 30% with the increase of miR-506 and miR-150 in leptin+ Panc-1 cells, respectively, as compared to leptin+ Panc-1 cells. However, palbociclib caused further increase of CD24 levels (****p < 0.0001. Although both miR-506 and miR-150 reduced CD24 levels in leptin−/+ MiaPaCa-2 cells, palbociclib treatment resulted in increased CD24 levels in each cell condition.

### 3.7. Mimic miR-150 significantly suppressed the CSC profile of pancreatic cancer cells by palbociclib cotreatment

**Figure 6:**
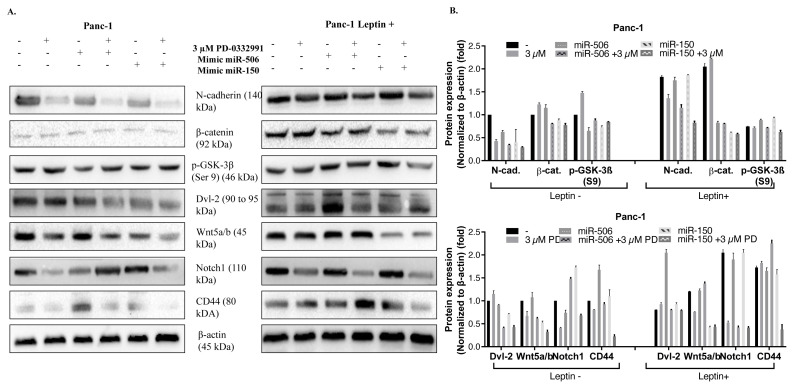

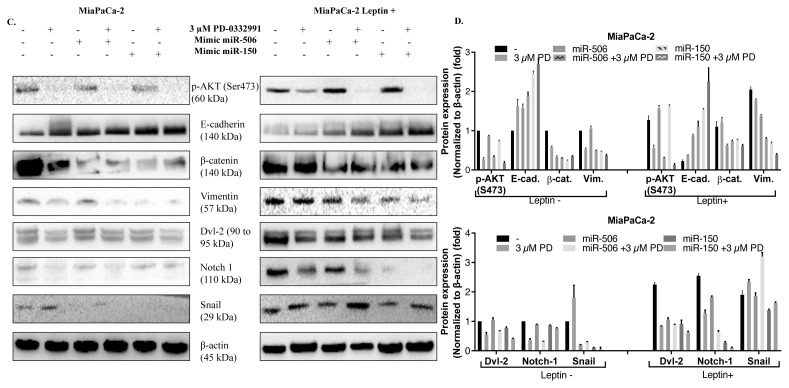
Leptin-induced Notch signaling was significantly inhibited by palbociclib and miR-150 treatment in Panc-1 and MiaPaCa-2 cells. **A–B.** The expression levels of N-cadherin, β-catenin, p-GSK3β (S9), Dvl-2, Wnt5a/b, Notch1, and CD44 were analyzed by western blotting in Leptin+/− Panc-1 cells that were transfected with miR-506 or miR-150 following palbociclib treatment (3 μM PD). **C–D.** The expression levels of p-Akt (Ser473), E-cadherin, β-catenin, Vimentin, Dvl-2, Notch1, and Snail were analyzed by western blotting in Leptin+/− MiaPaCa-2 cells that were transfected with miR-506 or miR-150 following palbociclib treatment (3 μM PD).

It has been observed that tumor formations can be suppressed by the increase in miR-506 and miR-150 expressions in leptin− and leptin+ cells, and they create a synergistic response in inhibiting cell proliferation with palbociclib. Therefore, after miR-506 and miR-150 transfection, total protein isolation was performed from leptin−/+ cells in the presence and absence of palbociclib. Then the expression levels of N-cadherin, β-catenin, p-GSK3β (S9), Dvl-2, Wnt5a/b, Notch1, and CD44 were determined in Panc-1 cells by western blotting (Figures 6A and 6B). Thirty micrograms of total protein lysate was run and transferred in 10%–12% SDS-PAGE under the same conditions and time. The western blot resulting images were acquired in Panc-1 and Panc-1 leptin+ cells at the same exposure times in order to make comparisons for the same primers. Accordingly, N-cadherin expression was decreased with palbociclib and more with miR-506 and miR-150 mimic transfection. N-cadherin level was higher in Leptin+ Panc-1 cells but decreased with palbociclib treatment. In particular, miR-150 mimic and palbociclib provided a significant decrease in N-cadherin levels. As expected, the β-catenin level was expressed higher in Panc-1 leptin+ cells. Although this level could not be decreased with palbociclib, increased -expression level of miR-506 or miR-150 decreased β-catenin levels. In fact, a significant reduction in β-catenin level was observed with miR-150 mimic and palbociclib treatment. The level of p-GSK3β decreased with increased expression of miR-506 and miR-150 in Panc-1 cells. Thus, it was thought that they might have increased the activation of GSK3β. Inhibition of GSK3β in leptin+ cells were an expected result, but treatment of palbociclib specifically with the miR-150 mimic inhibited GSK3β phosphorylation. Dvl-2 is a signaling molecule that functions in the Wnt signaling pathway highly expresses in pancreatic cancer cells. Dvl-2 level in Panc-1 cells did not change much with palbociclib treatment, but there was a significant decrease in Dvl-2 level with miR-506 and miR-150 mimic and palbociclib treatment. An increase in Dvl-2 level with miR-506 mimic in Leptin+ Panc-1 cells was noted. Wnt5a/b expression was decreased in leptin −/+ Panc-1 cells treated with palbociclib. Although, an increase in miR-506 expression did not make a difference in Wnt5a/b expression, an increase in a miR-150 level decreased Wnt5a/b expression. Notch-1, as the target molecule of leptin, was increased in Panc-1 leptin+ cells, but this increase was suppressed by palbociclib. CD44 expression was high in leptin+ cells, confirming the results of immunofluorescence and cell flow cytometry. In particular, the CD44 level decreased due to miR-150 mimic transfection and palbociclib treatments.

The expression levels of p-Akt (Ser473), E-cadherin, β-catenin, Vimentin, Dvl-2, Notch1 and Snail were determined in leptin−/+ MiaPaCa-2 cells (Figures 6C and 6D). Akt Ser473 phosphorylation is related to the activation of Akt and was inhibited by palbociclib treatment in MiaPaCa-2 cells. miR-506 and miR-150 mimic suppressed Akt phosphorylation. However, miR-506 and miR-150 alone caused an increase in mimic p-Akt, but palbociclib completely suppressed this increase in leptin+ cells. E-cadherin levels were lower in Leptin+ MiaPaCa-2 cells when compared with leptin-cells. Forced expression of miR-150 or miR-506 significantly upregulated the E-cadherin levels and combination with Palbociclib further increased the E-cadherin levels. The level of β-catenin was decreased in MiaPaCa-2 cells treated with palbociclib, but palbociclib did not make an effective difference on β-catenin in leptin+ cells. mimic miR-506 and miR-150 provided a significant reduction in β-catenin expression. The expression levels of Vimentin, Dvl-2, Notch and Snail were found to be expressed more in leptin+ cells, and when miR-150 expression increased their levels significantly decreased in both leptin− and leptin+ MiaPaCa-2 cells. Both mimic miR-506 and miR-150 synergistically reduced the expression levels of mesenchymal markers by palbociclib treatment.

## 4. Discussion

Obesity is a risk factor for pancreatic cancer presenting the increased plasma leptin level in and activating different signaling targets such as matrix-metalloproteinase-13 signaling resulting in increased angiogenesis (Yeh et al., 2009). In correlation with obesity, the modulated leptin signaling was a factor for PC that promoted cell proliferation by activating STAT3 and PI3K/Akt signals. We suggested that leptin mediated increased cell proliferation in PC drives aggressive phenotype of tumorigenesis potential. It is noteworthy that Notch is a contributing mechanism for colony formation mediated by leptin-induced changes in Wnt/β-catenin signaling. The induction of 3D forms of cells was apparent following leptin treatment at 40 ng/mL to mimic serum leptin concentration of obese patients (Buettner et al., 2006). Similarly, in vitro treatment of recombinant leptin to pancreatic cancer cells caused a significant increase in p-Akt in Panc-02 and Panc-1 pancreatic cancer cells, but MiaPaCa-2 cells did not activate p-Akt in response to leptin regarded to the short and long forms of the leptin receptor in cells (Mendonsa et al., 2015). It has been shown that tumor formations are cell-dependent in Panc-1 and MiaPaCa-2 xenograft models after leptin applications. Leptin acts as an mTOR activator and triggers intracellular protein and fat synthesis; however, our results are compatible with the literature in that leptin increases p-p70S6K and p-S6 activity in MiaPaCa-2 cells (Fazolini et al., 2015; Rencuzogulları et al., 2021). Activation of cell survival signals triggered by leptin was blocked by palbociclib treatment in Panc-1 and MiaPaCa-2 cells (Rencuzogullari et al., 2021). However, when each cell was compared with its own control group, Akt/mTOR and hedgehog and Wnt/β-catenin signal members were found to be high despite the treatment of palbociclib to leptin+ cells. In addition, CD133 level increased in MiaPaCa-2 cells treated with leptin, but remained unchanged in Panc-1 cells. CD133+ cells have active Wnt/β-catenin signaling and are in the migration stage when the rate of CD133+ cells is 9% in pancreatic tumors (Gzil et al., 2019). In this case, palbociclib and miRNA expressions targeting these signals were modulated suppressed the activation of these signaling pathways that provide cancer stem cell renewal and proliferation.

The effects of palbociclib on cell viability, EMT process, and stem cell markers by modulating miRNA expressions in Panc-1, MiaPaCa-2, and Capan-2 cells are discussed for each miRNA, respectively.

It has been determined that miR-506 is expressed at a very low level in PC cells due to hypermethylation in the miR-506 promoter region in PC cases (Li et al., 2016). Silencing of miR-506 due to hypermethylation has been a factor that increases the pathogenesis and progression of PC and causes poor prognosis. Palbociclib-mediated upregulation of miR-506 in Panc-1 and MiaPaCa-2 cells were similar to previous findings. In ovarian cancer, miR-506 is defined as an EMT inhibitor that inhibits cell migration and invasion; simultaneously, low level of miR-506 expression in tumor tissues in clinical studies showed a significant correlation with poor prognosis (Sun et al., 2015). Similar results have been demonstrated in cervical, breast, and gastric cancer, suggesting that miR-506 acts as a tumor suppressor in these cancer types (Arora et al., 2013; Wen et al., 2015). However, increased expression of miR-506 in melanomas and colon cancer promoted cell proliferation and acted as an oncogene as it caused drug resistance (Tong et al., 2011). In addition, miR-506 is associated with various biological behaviors through regulation of different target genes. Recent studies have shown that miR-506 inhibits TGFβ-induced EMT by targeting SNAI2 and suppressing vimentin and N-cadherin expression in ovarian cancer (Tong et al., 2011). In addition, miR-506 has been implicated in antiproliferative functions by regulating CDK6 and Gli3 target genes. These diverse observations of the roles of miR-506 are not surprising because the function of miRNAs and their target preferences can be highly dependent on the cellular and disease context. In a study in PC cells, regain of miR-506 expression induced cell cycle arrest at the G1/S transition and increased apoptosis and chemosensitivity. In the same study, it was found that miR-506 targets Akt and suppresses cell growth in vitro and in vivo (Li et al., 2016). In our study, forced expression of miR-506 expression in each cell line increased the effect of palbociclib to suppress EMT and tumorigenesis, and reduce CD44 and CD24 expressions. The additive effect of miR-506 to palbociclib treatment was more effective in MiaPaCa-2 cells. The CSC profile induced by leptin was inhibited by the increase of miR-506 expression and increased the sensitivity of leptin−/+ Panc-1 and MiaPaCa-2 cells to palbociclib. Therefore, it was thought that decreased miR-506 expression promoted the tumorigenesis of pancreatic cancer, while induced miR-506 expression increased the sensitivity of pancreatic cancer cells to palbociclib.

miR-150 has been identified as an oncogene or a tumor suppressor in various types of cancer (Chen et al., 2013; Zhang et al., 2018). A decrease in miR-150 was seen in most lymphohematopoietic tumors; however, increased miR-150 has been reported in gastric cancer and colorectal cancers metastases ( Wu et al., 2010; Li et al., 2018). The opposing effects of miR-150 on malignant tumors can be attributed to how its expression changes depending on cell characteristics. To date, reports on the role of miR-150 in the tumorigenesis of pancreatic cancers have been limited. It has been determined that miR-150 is expressed at very low levels in pancreatic cancer tumor tissues (Srivastava et al., 2011). According to the results obtained in clinical studies from PC tissue samples, it was determined that miR-150-positive individuals had a longer life expectancy than miR-150-negative individuals. miR-150 expression has been associated with tumor cell differentiation, stage of disease, lymph node metastasis, neural invasion, and distant metastasis in PC patients. c-Myc and Muc4, which are highly expressed in PC tissues and are related to aggression and metastasis, are among the target genes of miR-150 (Srivastava et al., 2011). Accordingly, it is an important outcome that c-Myc expression was reduced by palbociclib in our study (Rencuzogulları et al., 2020). In this study, the expression of β-catenin, which is among the targets of miR-150, did not show any significant difference when palbociclib was treated, especially in MiaPaCa-2 cells. In addition, while the nuclear localization of β-catenin was increased in MiaPaCa-2 cells treated with palbociclib; it was decreased in Panc-1 cells (Rencuzogulları et al., 2020). In relation to this, when miR-150 expression was examined in MiaPaCa-2 cells, it was determined that palbociclib caused a decrease in miR-150 expression. When miR-150 expression was increased in MiaPaCa-2 cells, β-catenin expression was significantly decreased. Moreover, the p-GSK3b (Ser9), Snail, Notch and Wnt5a/b level decreased through increased expression of miR-150 and palbociclib treatment in each cell line. It might be concluded that both miR-150 mimic and palbociclib treatment suppressed Wnt/b-catenin signaling mechanism. Tumorsphere formation induced by leptin in Panc-1 and MiaPaCa-2 cells was also suppressed when miR-150 expression was increased, increasing the sensitivity of cells to palbociclib (Rencuzogullari et al., 2021). In summary, miR-150 induced different responses depending on the cell type in the levels of tumor-initiating factors CD133 and CD24. In conclusion, miR-150 and palbociclib induced a synergistic response in inhibiting pancreatic cancer cell viability and migration.

miR-208 is highly expressed in cancer tissues as an oncomiR targeting E-cadherin which was increased following palbociclib treatment when the immunoblot results were compared (Rencuzogulları et al., 2020). However, in the migration analysis, it was determined that the migration rate of MiaPaCa-2 cells was higher in direct proportion to the increase of miR-208 expression in MiaPaCa-2 cells with palbociclib application, compared to Panc-1 cells. Therefore, by inhibiting miR-208 expression, palbociclib was found more effective in suppressing the migration potential of MiaPaCa-2 cells.

As a result, palbociclib suppressed the CSC profile triggered by leptin treatment, suppressing both tumorsphere structures and target signaling pathways of leptin, thereby breaking the resistance mechanism in Panc-1 and MiaPaCa-2 cells. miRNA expressions altered by miR-506, miR-150 mimic and anti-miR-208 treatments increased the sensitivity of Panc-1 and MiaPaCa-2 leptin−/+ cells to palbociclib. In accordance with the literature, the downregulation of miR-506 and miR-150, which are tumor suppressors, and upregulation of miR-208, an oncomiR, are the results that predict that these miRNAs can be used as a biomarker in PC cases with further studies. The fact that PC consists of heterogeneous cell groups is a parameter that limits the treatment process. For this reason, it has been confirmed in our study that monotherapies are insufficient in PC treatment, but the use of an inhibitor together increases the success rate. In the future, in vivo studies on developing miRNA therapies with palbociclib are planned. Two of the main areas of focus in the developing of miRNA therapeutics are to increase the in vivo stability of therapeutic RNA molecules and design optimal delivery systems for disease-specific release with minimal toxicity.

Figure S1 Effect of palbociclib on cell cycle phases in Panc-1 and MiaPaCa-2 cells transfected with miR-506, miR-150 mimic and anti-miR-208. Cells stained with PI were analyzed by reading 104 cells in flow cytometry. Data represented by histogram analysis are the mean ± Std.Dev. of two separate experiments. * p<0.05, **** p <0.0001
