# Supplementary figures and images for: Palbociclib suppresses the cancer stem cell properties and cell proliferation through increased levels of miR-506 or miR-150 in Panc-1 and MiaPaCa-2 cells
Source: Turk J Biol. 2022 Jul 18;46(5):342–60. doi: 10.55730/1300-0152.2622 (PMC10387936; doi:10.55730/1300-0152.2622)

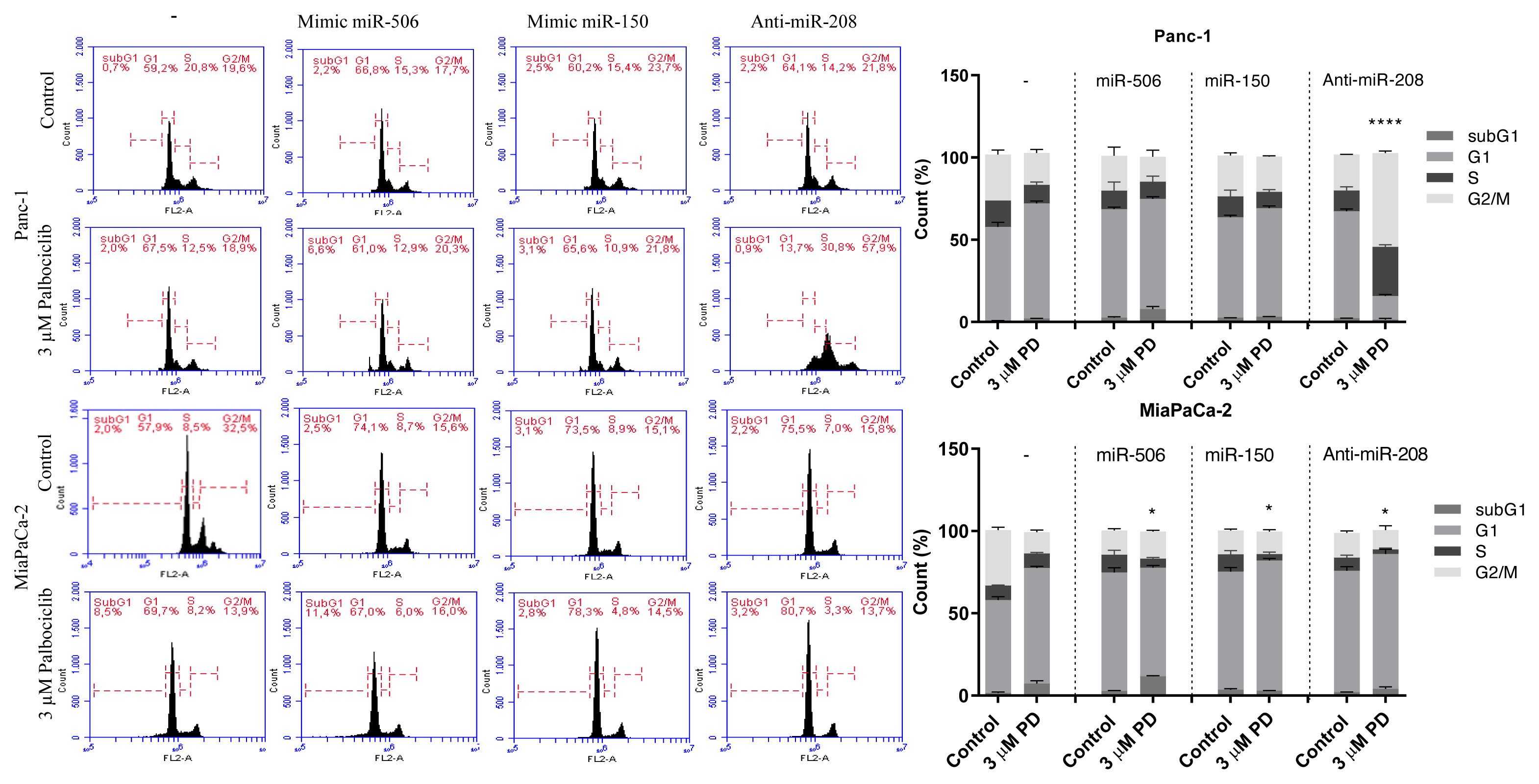

Supplement: Figure S1 — Effect of palbociclib on cell cycle phases in Panc-1 and MiaPaCa-2 cells transfected with miR-506, miR-150 mimic and anti-miR-208. Cells stained with PI were analyzed by reading 104 cells in flow cytometry. Data represented by histogram analysis are the mean ± Std.Dev. of two separate experiments. * p<0.05, **** p <0.0001 [file turkjbiol-46-5-342s1.tif]
